# Comparison of the acute outcome of two cryoballoon technologies for pulmonary vein isolation: An updated systematic review and meta-analysis

**DOI:** 10.1016/j.ijcha.2022.101115

**Published:** 2022-09-05

**Authors:** Amira Assaf, Rohit E. Bhagwandien, Tamas Szili-Torok, Sing-Chien Yap

**Affiliations:** Department of Cardiology, Erasmus MC, University Medical Center Rotterdam, Rotterdam, the Netherlands

**Keywords:** Atrial fibrillation, Cryoballoon, Cryoablation, Pulmonary vein isolation

## Abstract

Initial experience suggests that the POLARx cryoballoon system (Boston Scientific) has a similar procedural efficacy and safety as Arctic Front Advance Pro (AFA-Pro, Medtronic). We performed an updated systematic review and meta-analysis comparing POLARx and AFA-Pro. Embase, MEDLINE, Web of Science, Cochrane, and Google Scholar databases were searched until 12/01/2022 for studies comparing POLARx versus AFA-Pro in patients undergoing pulmonary vein (PV) isolation for AF. A total of 8 studies, involving 1146 patients from 11 European centers were included (POLARx n = 317; AFA-Pro n = 819). There were no differences in acute PV isolation, procedure time, fluoroscopy time, ablation time, minimal esophageal temperature, and risk of phrenic nerve palsy or thromboembolic events. Balloon nadir temperatures were lower for POLARx in all PVs. Compared with AFA-Pro, POLARx had a higher rate of first freeze isolation in the left inferior PV (LIPV) (odds ratio [OR]: 2.60; 95 % confidence interval [CI]: 1.06 to 6.43; P = 0.04), higher likelihood of time-to-isolation (TTI) recording in LIPV (OR: 2.91; 95 % CI: 1.54 to 5.49; P = 0.001) and right inferior PV (OR: 3.23; 95 % CI: 1.35 to 7.74; P = 0.008). In contrast, the TTI in LIPV was longer with POLARx in comparison to AFA-Pro (mean difference: 7.61 *sec*; 95 % CI 2.43 to 12.8 *sec*; P = 0.004). In conclusion, POLARx and AFA-Pro have a similar acute outcome. Interestingly, there was a higher rate of TTI recording in the inferior PVs with POLARx. This updated *meta*-analysis provides new safety data on esophageal temperature and thromboembolic events.

## 1 Introduction

Cryoballoon ablation has demonstrated to be as effective and safe as radiofrequency ablation for achieving pulmonary vein isolation (PVI). [1], [2], [3], [4], [5], [6], [7] The main advantages of the cryoballoon are the shorter procedure duration and relatively homogenous post-ablation outcomes. [1], [2], [3], [4], [5], [6], [7] The POLARx cryoablation system (Boston Scientific, Marlborough, MA, USA) was introduced in May 2020 and several observational studies reported their initial experience with this novel cryoballoon. [8], [9], [10], [11] In June 2021, we performed a *meta*-analysis of 4 clinical studies which demonstrated that POLARx had a similar procedural efficacy and safety in comparison to Arctic Front Advance Pro (AFA-Pro) (Medtronic, Minneapolis, MN, USA) despite a lower balloon nadir temperature. [12] After this publication, other centers has published their experience with POLARx. [13], [14], [15], [16] Therefore, we performed an updated systematic review and *meta*-analysis to confirm the robustness of the results of our previous *meta*-analysis. In addition, new outcome variables were evaluated such as time-to-isolation (TTI), likelihood of TTI recordings, minimal esophageal temperature, and thrombo-embolic events. As the POLARx cryoablation system becomes more widely adopted, we believe that this updated *meta*-analysis provides the most recent insights in the performance of the POLARx cryoablation system in comparison to AFA-Pro.

### 1.1 Aim of the study

The aim of this updated comprehensive *meta*-analysis was to compare differences in acute outcome between POLARx and AFA-Pro in patients with AF undergoing PVI.

## 2 Methods

### 2.1 Search strategy and study selection

This meta-analysis was performed in accordance with the Preferred Reporting Items for Systematic Reviews and meta-Analysis literature search extension (PRISMA-S) and meta-analysis Of Observational Studies in Epidemiology (MOOSE) checklists (Supplemental appendix A). [17], [18] The librarian-mediated systematic search strategy of our center was previously described. [19] The following electronic databases were searched on January 12, 2022: EMBASE (Ovid), MEDLINE (Ovid), Web of Science Core Collection (Web of Knowledge), Cochrane Central Register of Controlled Trials (Wiley) and Google Scholar. The search involved the following keywords: (“polarx” OR (“cryoablation” or “cryoballoon”) OR (“fourth-generation” or “4th-generation” or “4th-CB” or “CB4” or “CBG4” or “arctic front” or “AFA-Pro”)) AND (“pulmonary vein isolation” or “PVAI” or “PVI”). The complete search strategy per database is reported as supplemental material (Supplemental appendix B). We also searched ClinicalTrials.gov to identify ongoing trials. The search was limited to the English language and adult (18 years or older) human participants. All searches were limited to publications from 2019 to 2022 given that the POLARx cryoballoon was only commercially available in May 2020. Reference lists of included studies were manually screened to identify additional studies.

### 2.2 Eligibility criteria

The studies included fulfilled the following criteria: 1) patients with paroxysmal and/or persistent AF undergoing PVI with a cryoballoon; 2) comparison of POLARx cryoballoon with AFA-Pro cryoballoon; and 3) reported outcome data including but not limited to acute PVI success, procedure time, fluoroscopy time, ablation time, balloon nadir temperature, first freeze isolation, TTI recording, TTI, minimal esophageal temperature, phrenic nerve palsy (PNP) and stroke/transient ischemic attack (TIA). The following exclusion criteria were used: conference abstracts, case reports, review articles, editorials, and letters to the editor. Two reviewers screened articles using EndNote for inclusion independently, retrieved potentially relevant articles, and determined their eligibility. [20] Disagreements were resolved through consensus, and consultation of a third reviewer if necessary.

### 2.3 Data abstraction, data extraction and quality assessment

The following baseline patient characteristics were extracted from each included study: age, sex, type of AF, hypertension, diabetes, coronary artery disease and left atrial size. Extracted outcome data at patient level included: acute PVI success, procedure time, fluoroscopy time, ablation time, occurrence of PNP and stroke/TIA. The following parameters was extracted per individual pulmonary vein (PV) when available: balloon nadir temperature, first freeze isolation, TTI recording, TTI and minimal esophageal temperature. No authors were contacted as all relevant variables could be extracted from the published article. The quality of studies used in the analysis was assessed using the Newcastle Ottawa scale. Two reviewers independently performed data extraction and assessed study quality. Disagreements were resolved through consensus, and consultation of a third reviewer if necessary.

### 2.4 Statistical analysis

For continuous outcome variables, the pooled mean difference (MD) and the corresponding 95 % confidence intervals (CI) were estimated using the inverse-variance method. If a study provided medians and interquartile ranges or ranges, we estimated the means and standard deviations (SD) using Wan et al.’s method for the purpose of this *meta*-analysis. [21] For categorical outcome variables, the pooled odds ratio (OR) and corresponding 95 % CI were estimated using Mantel-Haenszel random-effects model. [22] A random-effects model was chosen a priori on the basis of the anticipated heterogeneity in baseline characteristics. Two-sided P-value < 0.05 was considered statistically significant. The presence of statistical heterogeneity was evaluated by Cochran’s Q test *I^2^* statistic. Statistical analysis was performed using Review Manager (RevMan, version 5.4.1., Copenhagen, the Nordic Cochrane Centre, The Cochrane Collaboration, 2020).

## 3 Results

### 3.1 Search results and baseline characteristics

**Fig. 1:**
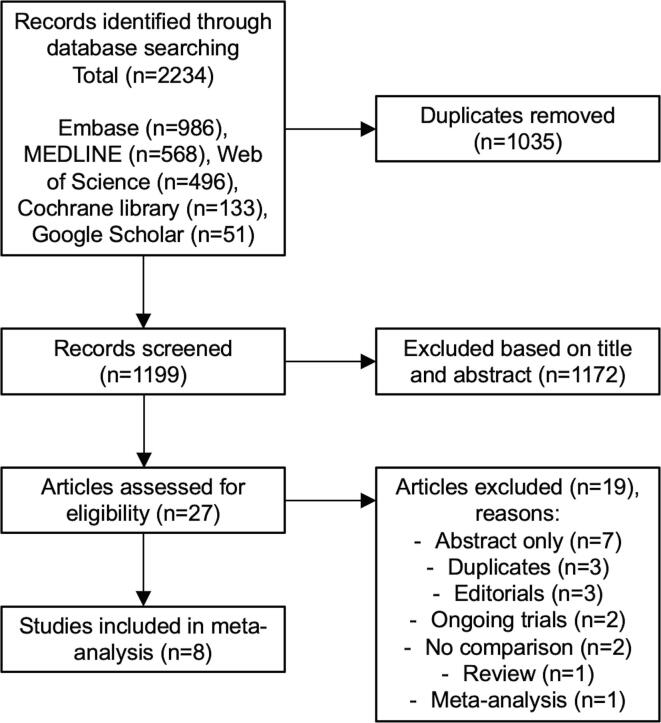
PRISMA flow chart for the selection of studies included in this *meta*-analysis.

Among 1199 unique citations, 27 citations were retrieved for full-text review. Following the review, a total of 8 studies met inclusion criteria (Fig. 1). [8], [9], [10], [11], [13], [14], [15], [16] All included studies were observational in design and found to be of good quality based on the Newcastle Ottawa scale (Supplemental table S1).

In total, 1146 patients from 11 European centers were included in the analysis of whom 317 and 819 patients underwent ablation with the POLARx and AFA-Pro system, respectively. The characteristics of the included studies are summarized in Table 1. Baseline patient characteristics among the included studies are shown in Table 2. The mean or median age of the patients ranged from 54 to 69 years and the proportion of males ranged from 52 % to 84 %. The proportion of patients with paroxysmal AF ranged from 36 % to 100 %.

**Table 1:** Studies included in the meta-analysis.

Study (year)	Country	Design	Freezing protocol	Bonus freeze	Number of patients in POLARx group	Number of patients in AFA-Pro group	Patient selection POLARx group	Patient selection AFA-Pro group
Creta et al. [8] (2021)	UK	Single-center	180 s	No	40	40	Consecutive cohort	Consecutive cohort
Guckel et al. [13] (2022)	Germany	Single-center	2x180 s	Yes	65	531	Consecutive cohort	Consecutive cohort from Jan 2013 to Aug 2021
Knecht et al. [14] (2021)	Switzerland	Multi-center	180–240 s	No	40	40	Consecutive cohort	Consecutive cohort
Kochi et al. [9] (2021)	Italy	Single-center	180–300 s	No	20	50	Consecutive cohort from Aug to Oct 2020	Consecutive cohort from Oct 2018 to Feb 2019
Mojica et al. [15] (2021)	Belgium	Single-center	180 s	Yes*	30	30	Consecutive cohort from Mar to Oct 2020	Propensity-matched cohort
Moser et al. [16] (2021)	Germany	Single-center	TTI + 120 s or 180 s	No	50	50	Consecutive cohort	Consecutive cohort
Tilz et al. [10] (2021)	Germany	Single-center	180–240 s**	Yes***	25	25	Consecutive cohort from Aug to Oct 2020	Consecutive cohort from May to July 2020
Yap et al. [11] (2021)	Croatia, Germany, Netherlands	Multi-center	180–240 s**	No	57	53	Consecutive cohort from May to Oct 2020	Consecutive cohort from May to Oct 2020

Abbreviations: PV, pulmonary vein; RCT, randomized controlled trial. * Only if TTI or balloon temperature −40 °C > 60 s. ** 180 s if TTI < 60 s, otherwise 240 s, *** Only if TTI > 60 s.

**Table 2:** Baseline characteristics of studies included in the *meta*-analysis.

Study	Age (years)		Male sex (%)		Paroxysmal AF (%)		Hypertension (%)		Diabetes (%)		Left atrial size	
	P	A	P	A	P	A	P	A	P	A	P	A
Creta et al. [8]	63	65	65 %	60 %	70 %	48 %	43 %	35 %	3 %	3 %	40 mm	38 mm
Guckel et al. [13]	65	63	69 %	75 %	66 %	53 %	57 %	41 %	11 %	15 %	–	–
Knecht et al. [14]	65	66	65 %	65 %	58 %	70 %	50 %	50 %	–	–	36 ml/m^2^	41 ml/m^2^
Kochi et al. [9]	63	61	60 %	84 %	95 %	94 %	60 %	30 %	5 %	6 %	36 ml/m^2^	33 ml/m^2^
Mojica et al. [15]	57	54	66 %	60 %	100 %	100 %	33 %	30 %	3 %	6 %	32 ml/m^2^	32 ml/m^2^
Moser et al. [16]	65	67	82 %	62 %	56 %	40 %	60 %	74 %	20 %	16 %	–	–
Tilz et al. [10]	68	69	52 %	68 %	48 %	36 %	80 %	72 %	12 %	12 %	25 ml/m^2^	29 ml/m^2^
Yap et al. [11]	61	64	58 %	68 %	75 %	76 %	32 %	59 %	5 %	6 %	41 mm	41 mm

Abbreviations: A, Arctic Front Advance Pro; AF, atrial fibrillation; P, POLARx.

### 3.2 Pooled analysis

**Fig. 2:**
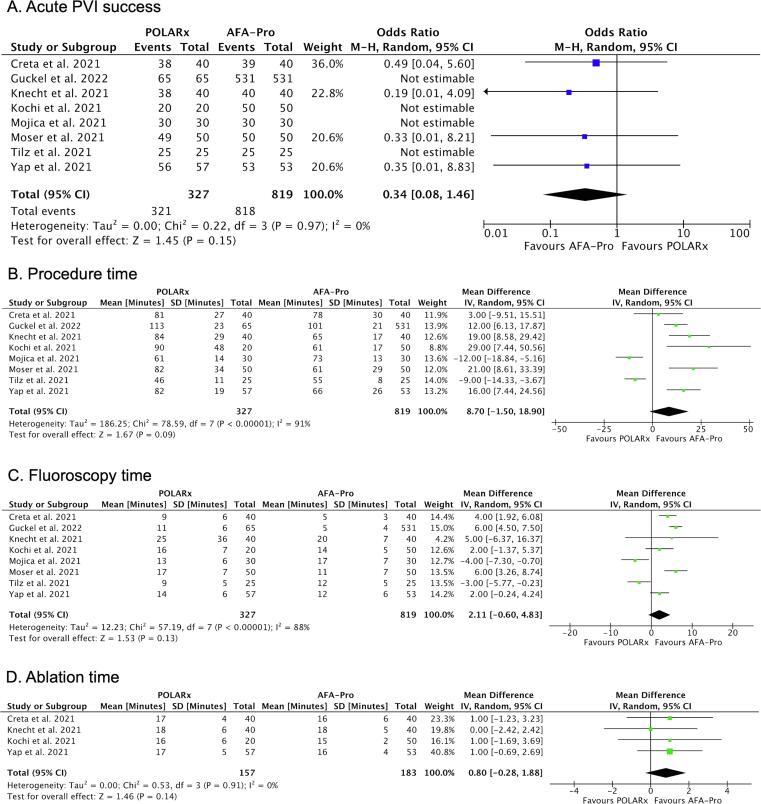
Forest plots of the pooled analysis demonstrating the effect of POLARx versus AFA-Pro on **procedural efficacy** in patients with AF. For acute PVI success, events and weighted odds ratios are presented. For continuous outcomes, mean, standard deviation and mean difference are presented. The horizontal line is the 95 % CI. The diamond shape is the estimate and the confidence interval of the estimate. A, acute PVI success; B, procedure time; C, fluoroscopy time; D, ablation time. Abbreviations: PVI = pulmonary vein isolation.

**Fig. 3:**
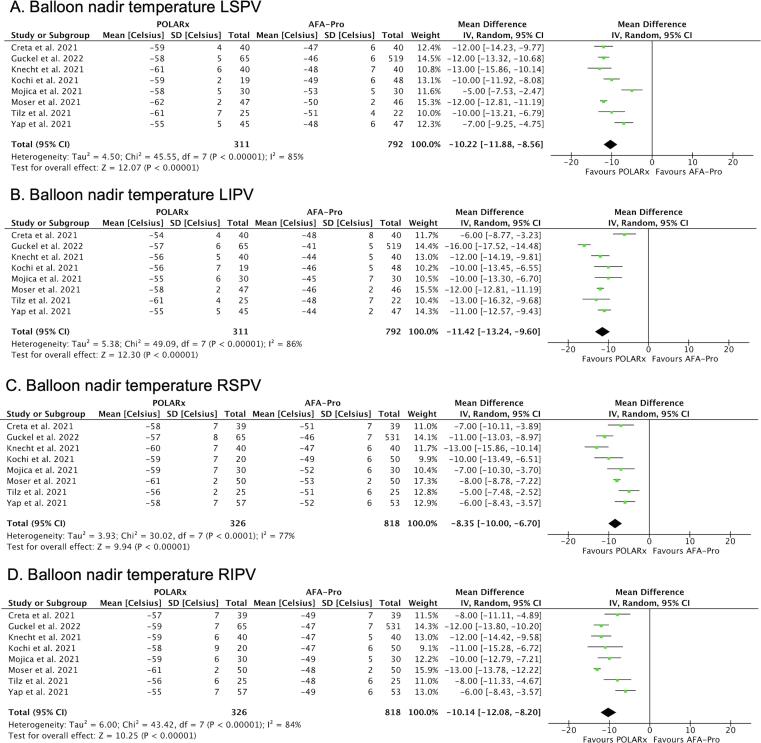
Forest plots of the pooled analysis demonstrating the effect of POLARx versus AFA-Pro on **balloon nadir temperature**. The data are presented as mean, standard deviation and mean difference. The horizontal line is the 95 % CI. The diamond shape is the estimate and the confidence interval of the estimate. Abbreviations: LIPV, left inferior pulmonary vein; LSPV, left superior pulmonary vein; RIPV, right inferior pulmonary vein, RSPV, right superior pulmonary vein.

There was no difference between POLARx and AFA-Pro in the rate of acute PVI, procedure time, fluoroscopy time and ablation time (Fig. 2). In comparison to AFA-Pro, the balloon nadir temperatures was lower with POLARx for all individual PVs (Fig. 3): left superior PV (LSPV) (MD: −10.22 °C; 95 % CI: −11.88 to −8.56; P < 0.001); left inferior PV (LIPV) (MD: −11.42 °C; 95 % CI: −13.24 to −9.60; P < 0.001); right superior PV (RSPV) (MD: −8.35 °C; 95 % CI: −10.00 to −6.70; P < 0.001); and right inferior PV (RIPV) (MD: −10.14 °C; 95 % CI: −12.08 to −8.20; P < 0.001).

**Fig. 4:**
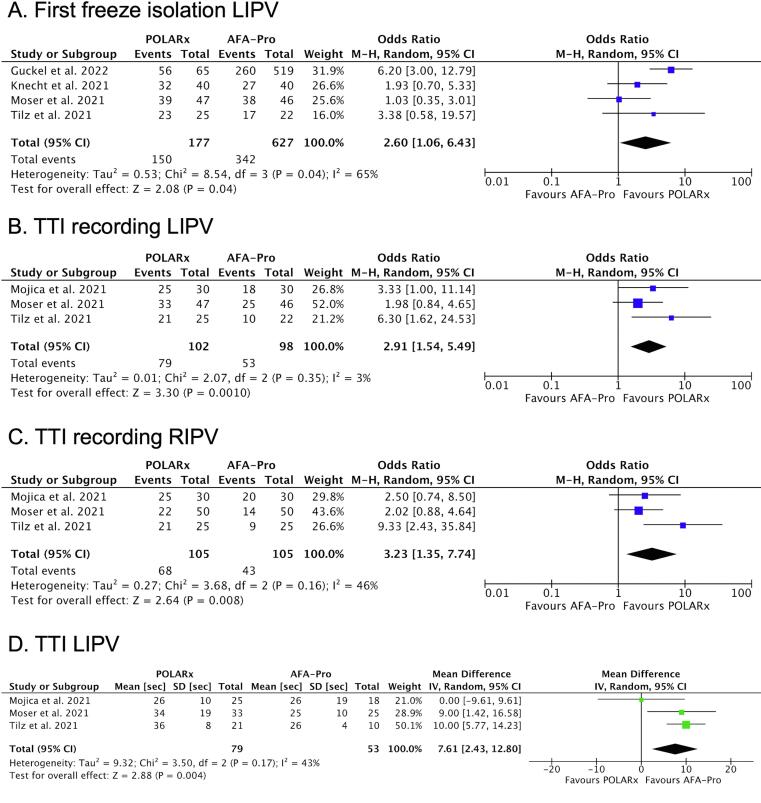
Forest plots of the pooled analysis demonstrating the effect of POLARx versus AFA-Pro on **first freeze isolation LIPV**, **TTI recording inferior PVs**, and **TTI LIPV**. Events and weighted odds ratios are presented, except for TTI, where mean, standard deviation and mean difference are presented. The horizontal line is the 95 % CI. The diamond shape is the estimate and the confidence interval of the estimate. Abbreviations: LIPV, left inferior pulmonary vein; RIPV, right inferior pulmonary vein; TTI, time to isolation.

**Fig. 5:**
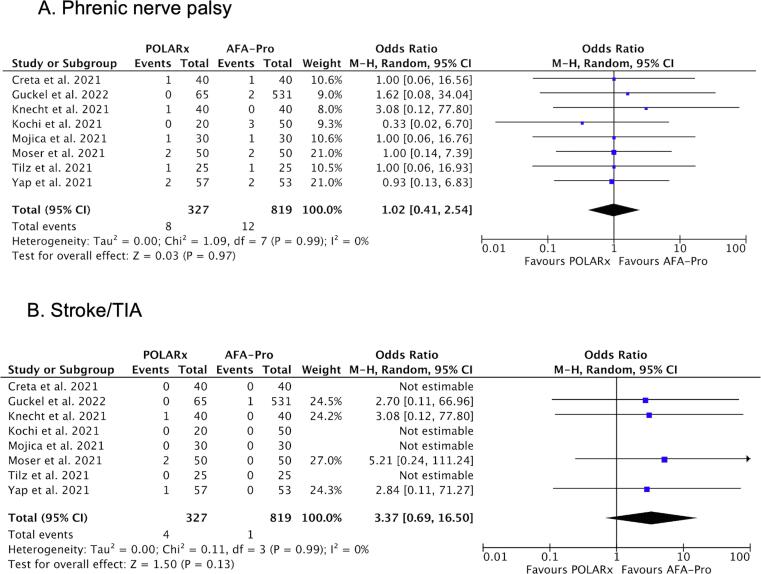
Forest plot of the pooled analysis demonstrating the effect of POLARx versus AFA-Pro on **periprocedural complications**. The data are presented as events and weighted odds ratios. The horizontal line is the 95 % CI. The diamond shape is the estimate and the confidence interval of the estimate.

POLARx had a higher likelihood of achieving first freeze isolation in the LIPV (OR: 2.60; 95 % CI: 1.06 to 6.43; P = 0.04) (Fig. 4A). The likelihood of first freeze isolation in the other PVs was similar between systems (Supplemental figure S1). POLARx was associated with a higher rate of TTI recording in the LIPV (OR: 2.91; 95 % CI: 1.54 to 5.49; P = 0.001) and in the RIPV (OR: 3.23; 95 % CI: 1.35 to 7.74; P = 0.008) (Fig. 4B and 4C). The rate of TTI recording in the superior PVs was similar between systems (Supplemental figure S2). When TTI could be recorded, the TTI in the LIPV was longer with POLARx in comparison to AFA-Pro (MD: 7.61 *sec*; 95 % CI 2.43 to 12.8 *sec*; P = 0.004) (Fig. 4D). The TTI in the other PVs was similar between systems (Supplemental figure S3). The minimal esophageal temperature per PV was similar between POLARx and AFA-Pro (Supplemental figure S4). Finally, there was no difference in the incidence of PNP and stroke/TIA between the two modalities (Fig. 5).

### 3.3 Sensitivity analysis

There was significant statistical heterogeneity (*I^2^* ≥ 50 %) for the outcomes of procedure time, fluoroscopy time, balloon nadir temperatures, first freeze isolation (except LSPV), TTI recording in superior PVs, TTI (except LIPV) and minimal esophageal temperature RIPV. For the outcomes of procedure time, fluoroscopy time and balloon nadir temperatures the between-study heterogeneity remained high (*I^2^* ≥ 50 %) with the sequential exclusion of studies. For the outcomes first freeze isolation RSPV, first freeze isolation RIPV, TTI recording RSPV and TTI RIPV, the between-study heterogeneity became < 50 % after the exclusion of a single study, however, the overall effect size did not change. Heterogeneity for the outcome of first freeze isolation LIPV was driven primarily by the study of Guckel et al. [13] After the exclusion of this study, no difference between was detected in the likelihood of first freeze isolation LIPV (P = 0.16) (Supplemental figure S5A). Heterogeneity for the outcomes of TTI recording LSPV, TTI LSPV and TTI RSPV was driven primarily by the study of Moser et al. [16] After the exclusion of this study, TTI recording LSPV and TTI LSPV became in favour of POLARx (P = 0.004 and P = 0.02, respectively) (Supplemental figure S5B and S5C). Furthermore, sensitivity analysis demonstrated that TTI RSPV became in favour of AFA-Pro after the exclusion of the study of Moser et al. (P < 0.001) (Supplemental figure S5D). [16] No funnel plots were constructed to examine publication bias due to the low number of included studies (<10). The power of the test would be too low to distinguish chance from real asymmetry.

## 4 Discussion

This updated *meta*-analysis demonstrates that POLARx and AFA-Pro have a similar procedural efficacy and safety in patients with symptomatic AF. In addition to our previous published *meta*-analysis, we provide new comparative data on TTI, rate of TTI recordings, and safety (minimal esophageal temperature and thrombo-embolic events). Interestingly, the rate of TTI recordings in the inferior PVs was higher with POLARx. Considering that a large, randomized trial is not expected soon, our data provide the most comprehensive periprocedural data comparing POLARx and AFA-Pro.

The use of cryoballoon technology to achieve PVI is effective, and it provides homogenous lesions with a low arrhythmogenic potential. [1], [2], [3], [4], [5], [6], [7] The Arctic Front cryoballoon has undergone multiple modifications, and the fourth generation AFA-Pro is currently the most widely used cryoballoon. The novel POLARx cryoballoon has many similarities with AFA-Pro (e.g., double-layer balloon, 28 mm balloon size, nitrous oxide cooling technology), but the inner balloon pressure is kept constant during the inflation and freezing phase. Thus, the inner balloon pressure of POLARx is lower than AFA-Pro during the freezing phase resulting in a more compliant balloon.

The novel cryoballoon characteristics of POLARx, in combination with the new features of the cryoconsole and steerable sheath, is associated with a learning curve effect. [11] Although the pooled estimate did not show a difference in procedure time and fluoroscopy time, the between-study heterogeneity for both was high (*I^2^* 91 % and 88 %, respectively). Only the study of Tilz et al. and Mojica et al. demonstrated a shorter procedure and fluoroscopy time with POLARx in comparison to AFA-Pro. [10], [15] Nevertheless, the results of our *meta*-analysis suggest that the novel POLARx cryoablation system only has a short learning curve in experienced cryoballoon centers and has a similar procedural efficacy as the established AFA-Pro.

The balloon nadir temperatures are lower with POLARx than AFA-Pro, which was already shown in the individual studies and our previous *meta*-analysis. [12] There was a high between-study heterogeneity, but the forest plots show that balloon nadir temperatures are lower with POLARx across all studies. It is important to realize that the measured inner balloon temperature is not equal to the surface balloon temperature. Many factors may affect the inner balloon temperatures, such as location of the thermocouple, efficacy of energy transfer to atrial tissue by thermoplastic balloon material, depth of balloon in the PV and/or balloon-tissue contact area. [8], [14].

Interestingly, the likelihood of a TTI recording in the inferior PVs was higher with POLARx in comparison to AFA-Pro. This may be related to the shorter distal tip of POLARx when the short tip version is used (5 versus 8 mm for POLARx and AFA-Pro, respectively). However, the prevalence of TTI recordings in the superior PVs was similar between POLARx and AFA-Pro. Another explanation may be that the Achieve circular mapping catheter is placed more distally in the inferior PVs with AFA-Pro in order to provide more balloon stability. When approaching the inferior PVs, the balloon tip is usually oriented downwards by curving the steerable sheath. During freezing, the balloon shape of AFA-Pro changes due to an increase in balloon pressure. To compensate for potential displacement of the balloon and to retain adequate PV occlusion, additional balloon stability is sometimes required by placing the circular mapping catheter more distally. Thus, the higher likelihood of TTI recording in the inferior PVs with POLARx may be due to the lower need to place the POLARMAP mapping catheter more distally to provide stability because the POLARx balloon does not change shape during the freezing phase. Furthermore, there was a lower TTI in the LIPV with AFA-Pro in comparison to POLARx. The faster isolation with AFA-Pro in the LIPV may be explained by using the “pull-down” maneuver during freezing or the use of increased forward push (to counteract balloon dislocation when starting freezing). Catheter manipulations during freezing and high forward push are not advised by the manufacturer of POLARx. Unfortunately, we could not extract detailed data on catheter handling from the individual studies, thus, abovementioned potential explanations for the shorter TTI in the LIPV with AFA-Pro are speculative and largely based on clinical observation.

After the introduction of a new technology, it is important to evaluate the safety of the device. PNP is a well-known complication of a cryoballoon procedure, and this is especially important considering the lower balloon nadir temperatures with POLARx. The current *meta*-analysis, including more patients, reconfirms that the incidence of PNP between POLARx and AFA-Pro is similar. [12] Furthermore, the minimal esophageal temperatures during freezing were similar between both cryoablation systems (Supplemental figure S4). Thus, it seems that the lower measured inner balloon nadir temperatures with POLARx does not translate to a lower balloon surface temperature in comparison to AFA-Pro. Two large registry data with the Arctic Front cryoablation system have shown that the incidence of persistent PNP is low (<0.5 %). [23], [24] We expect that PNP recovery will also occur in the majority of patients using POLARx, however, currently there is limited published data on the long-term outcome of acute PNP with this novel system. [25].

Finally, the risk of periprocedural stroke/TIA was similar between POLARx and AFA-Pro (OR: 3.37; 95 % CI: 0.69 to 16.50; P = 0.13). Nevertheless, a total of 4 events of stroke/TIA were reported in 327 patients (1.2 %). Yap et al. reported one patient with a transient left-sided hemiparesis due to a TIA with no demarcation of infarct area. [11] Moser et al. reported two patients with a periprocedural stroke with symptoms directly after the procedure. [16] Knecht et al. report one patient with periprocedural stroke due to air embolism. [14] The air embolism in this patient caused an initial left-sided hemiparesis with progression to coma 6 h after the procedure. The patient recovered with only minimal symptoms after 48 h of coma. After reports of air embolism, Boston Scientific issued an urgent field safety notification in April 2021 for the POLARSHEATH. The Instructions for Use were supplemented to highlight the risk of air embolism and to provide guidance to minimize the risk for air ingress when using the POLARSHEATH.

## 5 Study limitations

All studies included in this *meta*-analysis were observational studies, but they were of good quality based on the Newcastle Ottawa scale. Currently, one randomized controlled trial comparing POLARx and AFA-Pro for the treatment of paroxysmal AF is recruiting patients (NCT04704986, COMPARE-CRYO) (estimated study size 200 patients) but the results are not expected soon. Two large prospective single-arm studies, POLAR-ICE (NCT04250714) and FROZEN-AF (NCT04133168), will provide outcome data of POLARx but these trials do not provide head-to-head comparison between both cryoballoon technologies.

For some outcome parameters there was significant heterogeneity between studies, but to account for this we used a random-effects model a priori. Finally, we report only procedural outcome; thus, we do not have data on long-term outcome such as persistent PNP, PV stenosis, atrio-esophageal fistula and freedom from atrial arrhythmia. This limitation is inherent to the relatively recent introduction of the POLARx cryoballoon. Finally, it is important to note that the publications were from centers with extensive experience with cryoballoon procedures which limits generalizability of the data.

## 6 1. Conclusion

The acute outcome of POLARx is comparable to AFA-Pro, despite lower balloon nadir temperatures with POLARx. Interestingly, there was a higher rate of TTI recording in the inferior PVs with POLARx. This updated *meta*-analysis provides new safety data on minimal esophageal temperature and thromboembolic events.

Funding.

The authors did not receive support from any organization for the submitted work.

## Declaration of Competing Interest

The authors declare the following financial interests/personal relationships which may be considered as potential competing interests: SCY is a consultant for Boston Scientific and has received speaker honorarium from Medtronic. The other authors have no competing interests to declare that are relevant to the content of this article.

## References

[1] Kuck K.-H., Brugada J., Fürnkranz A., Metzner A., Ouyang F., Chun K.R.J., Elvan A., Arentz T., Bestehorn K., Pocock S.J., Albenque J.-P., Tondo C. (2016). Investigators ICE. Cryoballoon or Radiofrequency Ablation for Paroxysmal Atrial Fibrillation. N Engl J Med..

[2] Luik A., Radzewitz A., Kieser M., Walter M., Bramlage P., Hörmann P., Schmidt K., Horn N., Brinkmeier-Theofanopoulou M., Kunzmann K., Riexinger T., Schymik G., Merkel M., Schmitt C. (2015). Cryoballoon Versus Open Irrigated Radiofrequency Ablation in Patients With Paroxysmal Atrial Fibrillation: The Prospective, Randomized, Controlled, Noninferiority FreezeAF Study. Circulation..

[3] Buiatti A, von Olshausen G, Barthel P, et al. Hoppmann P. Cryoballoon vs. radiofrequency ablation for paroxysmal atrial fibrillation: an updated meta-analysis of randomized and observational studies. *Europace*. 2017;19:378-384. 10.1093/europace/euw262 27702864

[4] Andrade J.G., Champagne J., Dubuc M., Deyell M.W., Verma A., Macle L., Leong-Sit P., Novak P., Badra-Verdu M., Sapp J., Mangat I., Khoo C., Steinberg C., Bennett M.T., Tang A.S.L., Khairy P., Parkash R., Guerra P., Dyrda K., Rivard L., Racine N., Sterns L., Leather R., Seifer C., Jolly U., Raymond J.-M., Roux J.-F., Nault I., Sarrazin J.-F., Ramanathan K., Cheung C., Fordyce C., McKinney J., Luong C., Rizkallah J., Angaran P., Ha A., Glover B., Skanes A., Gula L. (2019). Investigators C-DS. Cryoballoon or Radiofrequency Ablation for Atrial Fibrillation Assessed by Continuous Monitoring: A Randomized Clinical Trial. Circulation..

[5] Fortuni F., Casula M., Sanzo A., Angelini F., Cornara S., Somaschini A., Mugnai G., Rordorf R., De Ferrari G.M. (2020). De Ferrari GM. Meta-Analysis Comparing Cryoballoon Versus Radiofrequency as First Ablation Procedure for Atrial Fibrillation. Am J Cardiol..

[6] Luik A., Kunzmann K., Hörmann P., Schmidt K., Radzewitz A., Bramlage P., Schenk T., Schymik G., Merkel M., Kieser M., Schmitt C. (2017). Cryoballoon vs. open irrigated radiofrequency ablation for paroxysmal atrial fibrillation: long-term FreezeAF outcomes. BMC Cardiovasc Disord..

[7] Providencia R., Defaye P., Lambiase P.D., Boveda S. (2017). Results from a multicentre comparison of cryoballoon vs. radiofrequency ablation for paroxysmal atrial fibrillation: is cryoablation more reproducible?. Europace..

[8] Creta A., Kanthasamy V., Schilling R.J., Rosengarten J., Khan F., Honarbakhsh S., Earley M.J., Hunter R.J., Finlay M. (2021). First experience of POLARx versus Arctic Front Advance: An early technology comparison. J Cardiovasc Electrophysiol..

[9] Kochi A.N., Moltrasio M., Tundo F., Riva S., Ascione C., Dessanai M.A., Pizzamiglio F., Vettor G., Cellucci S., Gasperetti A., Tondo C., Fassini G. (2021). Cryoballoon atrial fibrillation ablation: Single-center safety and efficacy data using a novel cryoballoon technology compared to a historical balloon platform. J Cardiovasc Electrophysiol..

[10] Tilz R.R., Meyer-Saraei R., Eitel C. (2021). Heeger CH. Novel Cryoballoon Ablation System for Single Shot Pulmonary Vein Isolation- The Prospective ICE-AGE-X Study. Circ J..

[11] Yap S.-C., Anic A., Breskovic T., Haas A., Bhagwandien R.E., Jurisic Z., Szili‐Torok T., Luik A. (2021). Comparison of procedural efficacy and biophysical parameters between two competing cryoballoon technologies for pulmonary vein isolation: Insights from an initial multicenter experience. J Cardiovasc Electrophysiol..

[12] Assaf A., Bhagwandien R., Szili‐Torok T., Yap S.-C. (2021). Comparison of procedural efficacy, balloon nadir temperature, and incidence of phrenic nerve palsy between two cryoballoon technologies for pulmonary vein isolation: A systematic review and meta-analysis. J Cardiovasc Electrophysiol..

[13] Guckel D., Lucas P., Isgandarova K., El Hamriti M., Bergau L., Fink T., Sciacca V., Imnadze G., Braun M., Khalaph M., Nölker G., Sommer P., Sohns C. (2022). News from the Cold Chamber: Clinical Experiences of POLARx versus Arctic Front Advance for Single-Shot Pulmonary Vein Isolation. J Cardiovasc Dev Dis..

[14] Knecht S., Sticherling C., Roten L., Badertscher P., Chollet L., Küffer T., Spies F., Madaffari A., Mühl A., Baldinger S.H., Servatius H., Osswald S., Reichlin T., Kühne M. (2022). Technical and procedural comparison of two different cryoballoon ablation systems in patients with atrial fibrillation. J Interv Card Electrophysiol.

[15] Mojica J, Lipartiti F, Al Housari M, et al. Chierchia GB. Procedural Safety and Efficacy for Pulmonary Vein Isolation with the Novel Polarx TM Cryoablation System: A Propensity Score Matched Comparison with the Arctic Front TM Cryoballoon in the Setting of Paroxysmal Atrial Fibrillation. *J Atr Fibrillation*. 2021;14:20200455. 10.4022/jafib.20200455 PMC8691321 34950358

[16] Moser F., Rottner L., Moser J., Schleberger R., Lemoine M., Münkler P., Dinshaw L., Kirchhof P., Reissmann B., Ouyang F., Rillig A., Metzner A. (2022). The established and the challenger: A direct comparison of current cryoballoon technologies for pulmonary vein isolation. J Cardiovasc Electrophysiol..

[17] Stroup D.F., Berlin J.A., Morton S.C., Thacker S.B. (2000). Meta-analysis of observational studies in epidemiology: a proposal for reporting. Meta-analysis Of Observational Studies in Epidemiology (MOOSE) group. JAMA..

[18] Rethlefsen M.L., Kirtley S., Waffenschmidt S., Ayala A.P., Moher D., Page M.J., Koffel J.B. (2021). Group P-S. PRISMA-S: an extension to the PRISMA Statement for Reporting Literature Searches in Systematic Reviews. Syst Rev..

[19] Bramer W.M., de Jonge G.B., Rethlefsen M.L., Mast F., Kleijnen J. (2018). A systematic approach to searching: an efficient and complete method to develop literature searches. J Med Libr Assoc..

[20] Bramer W.M., Milic J., Mast F. (2017). Reviewing retrieved references for inclusion in systematic reviews using EndNote. J Med Libr Assoc..

[21] Wan X., Wang W., Liu J., Tong T. (2014). Estimating the sample mean and standard deviation from the sample size, median, range and/or interquartile range. BMC Med Res Methodol..

[22] Borenstein M., Hedges L.V., Higgins J.P., Rothstein H.R. (2010). A basic introduction to fixed-effect and random-effects models for meta-analysis. Res Synth Methods..

[23] Heeger CH, Sohns C, Pott A, et al. Richard Tilz R. Phrenic Nerve Injury During Cryoballoon-Based Pulmonary Vein Isolation: Results of the Worldwide YETI Registry. *Circ Arrhythm Electrophysiol*. 2022;15:e010516. 10.1161/CIRCEP.121.010516 PMC8772436 34962134

[24] Mol D., Renskers L., Balt J.C. (2022).

[25] Anic A., Lever N., Martin A., Varma N. (2021). Acute safety, efficacy, and advantages of a novel cryoballoon ablation system for pulmonary vein isolation in patients with paroxysmal atrial fibrillation: initial clinical experience. Europace..

