# Supplementary material for: Comparison of the acute outcome of two cryoballoon technologies for pulmonary vein isolation: An updated systematic review and meta-analysis
Source: Int J Cardiol Heart Vasc. 2022 Sep 5;42:101115. doi: 10.1016/j.ijcha.2022.101115 (PMC9463571; doi:10.1016/j.ijcha.2022.101115)
Supplement: Supplementary figures and tables (multimedia component 1, supplementary data 1) [file mmc1.docx]

**Supplemental figure S1**. Forest plots of the pooled analysis demonstrating the effect of POLARx versus AFA-Pro on **proportion of first freeze isolation** per pulmonary vein. The data are presented as events and weighted odds ratios. The horizontal line is the 95% CI. The diamond shape is the estimate and the confidence interval of the estimate. Abbreviations: LIPV, left inferior pulmonary vein; LSPV, left superior pulmonary vein; RIPV, right inferior pulmonary vein, RSPV, right superior pulmonary vein.


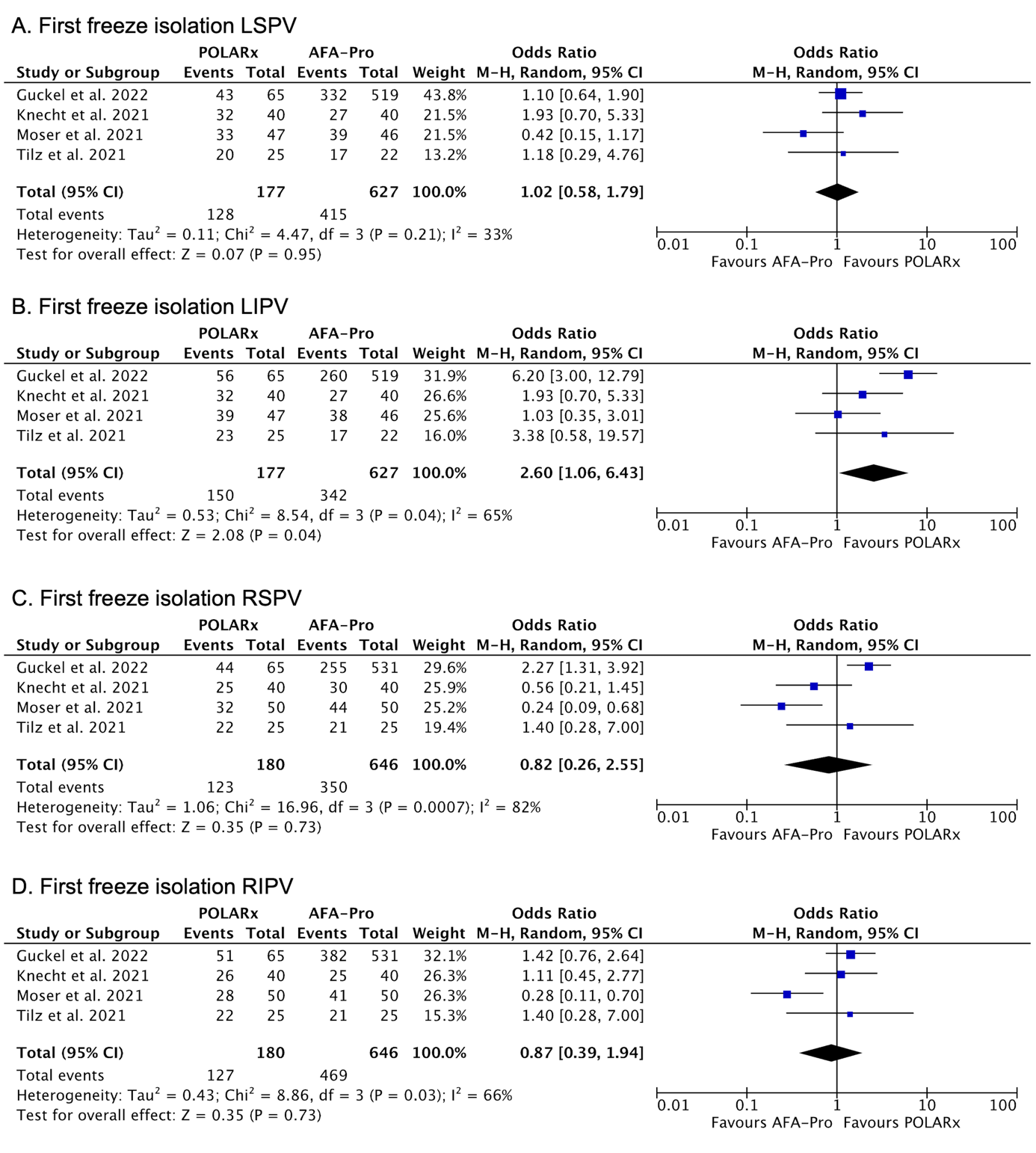


**Supplemental figure S2**. Forest plots of the pooled analysis demonstrating the effect of POLARx versus AFA-Pro on **proportion of TTI** recording per pulmonary vein. The data are presented as events and weighted odds ratios. The horizontal line is the 95% CI. The diamond shape is the estimate and the confidence interval of the estimate. Abbreviations: LIPV, left inferior pulmonary vein; LSPV, left superior pulmonary vein; RIPV, right inferior pulmonary vein, RSPV, right superior pulmonary vein.

**
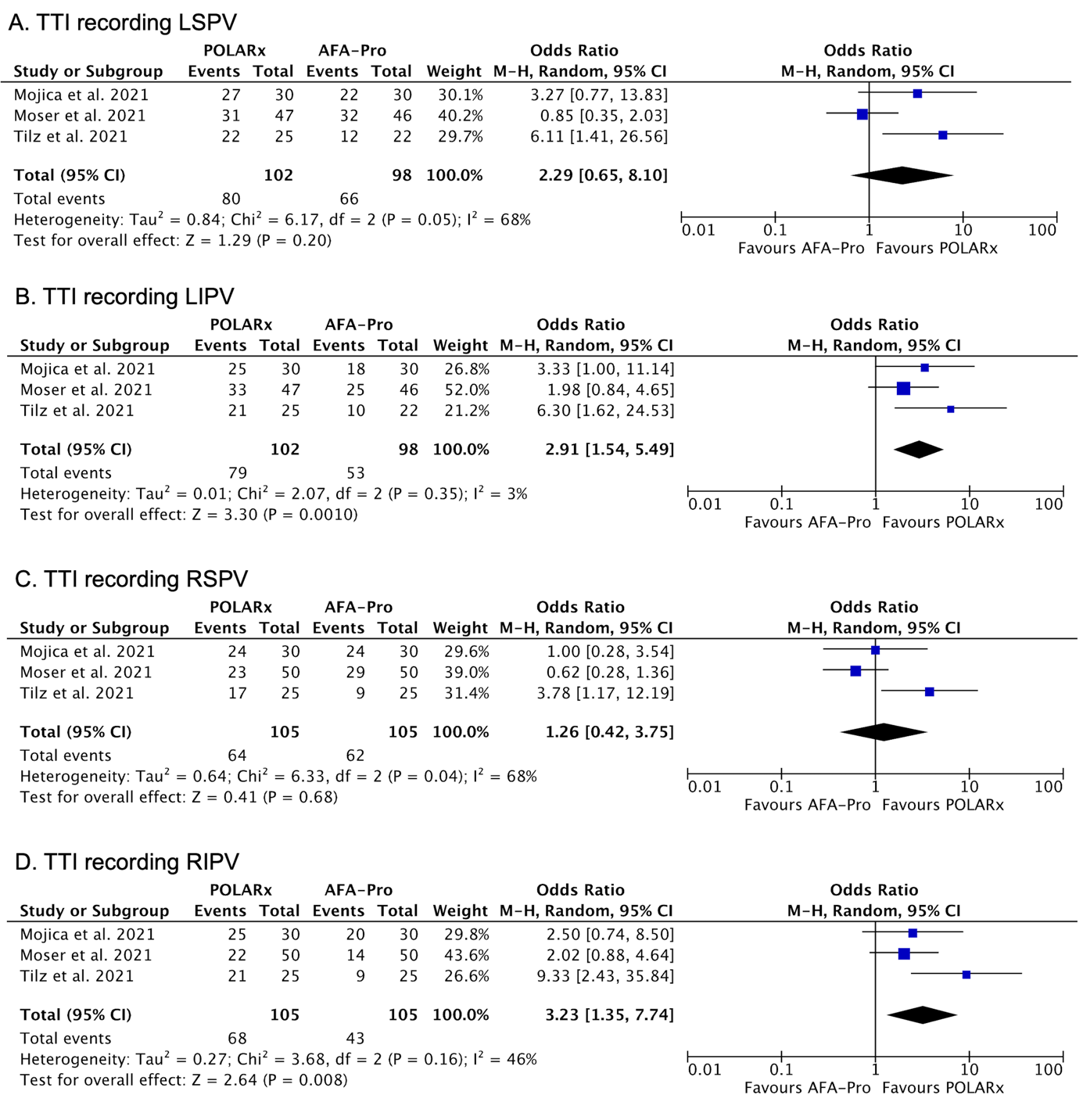
**

**Supplemental figure S3**. Forest plots of the pooled analysis demonstrating the effect of POLARx versus AFA-Pro on **time-to-isolation (TTI)** per pulmonary vein. The data are presented as mean, standard deviation and mean difference. The horizontal line is the 95% CI. The diamond shape is the estimate and the confidence interval of the estimate. Abbreviations: LIPV, left inferior pulmonary vein; LSPV, left superior pulmonary vein; RIPV, right inferior pulmonary vein, RSPV, right superior pulmonary vein.

**
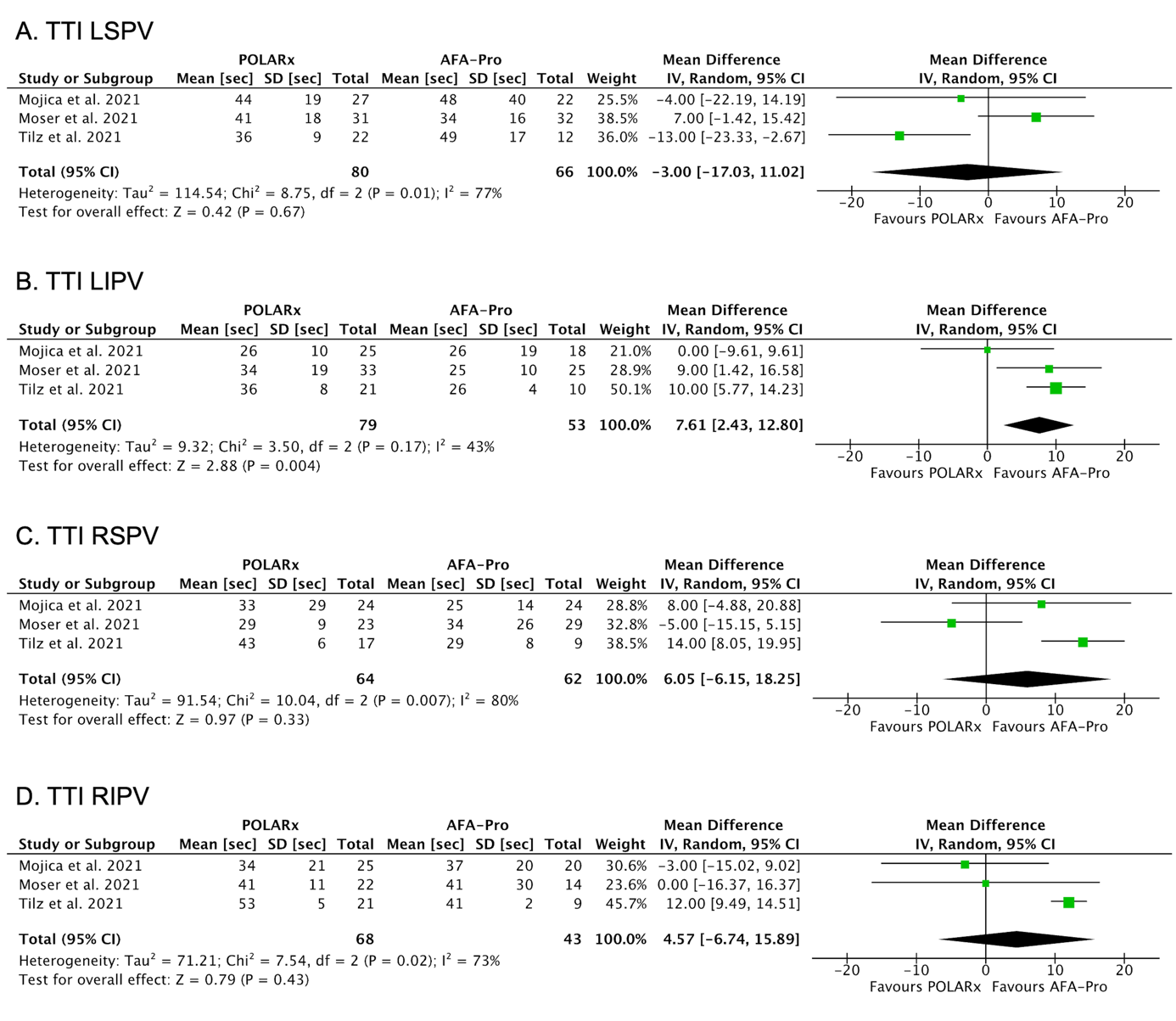
**

**Supplemental figure S4**. Forest plots of the pooled analysis demonstrating the effect of POLARx versus AFA-Pro on the **minimal esophageal temperature** per pulmonary vein. The data are presented as mean, standard deviation and mean difference. The horizontal line is the 95% CI. The diamond shape is the estimate and the confidence interval of the estimate. Abbreviations: LIPV, left inferior pulmonary vein; LSPV, left superior pulmonary vein; RIPV, right inferior pulmonary vein, RSPV, right superior pulmonary vein.


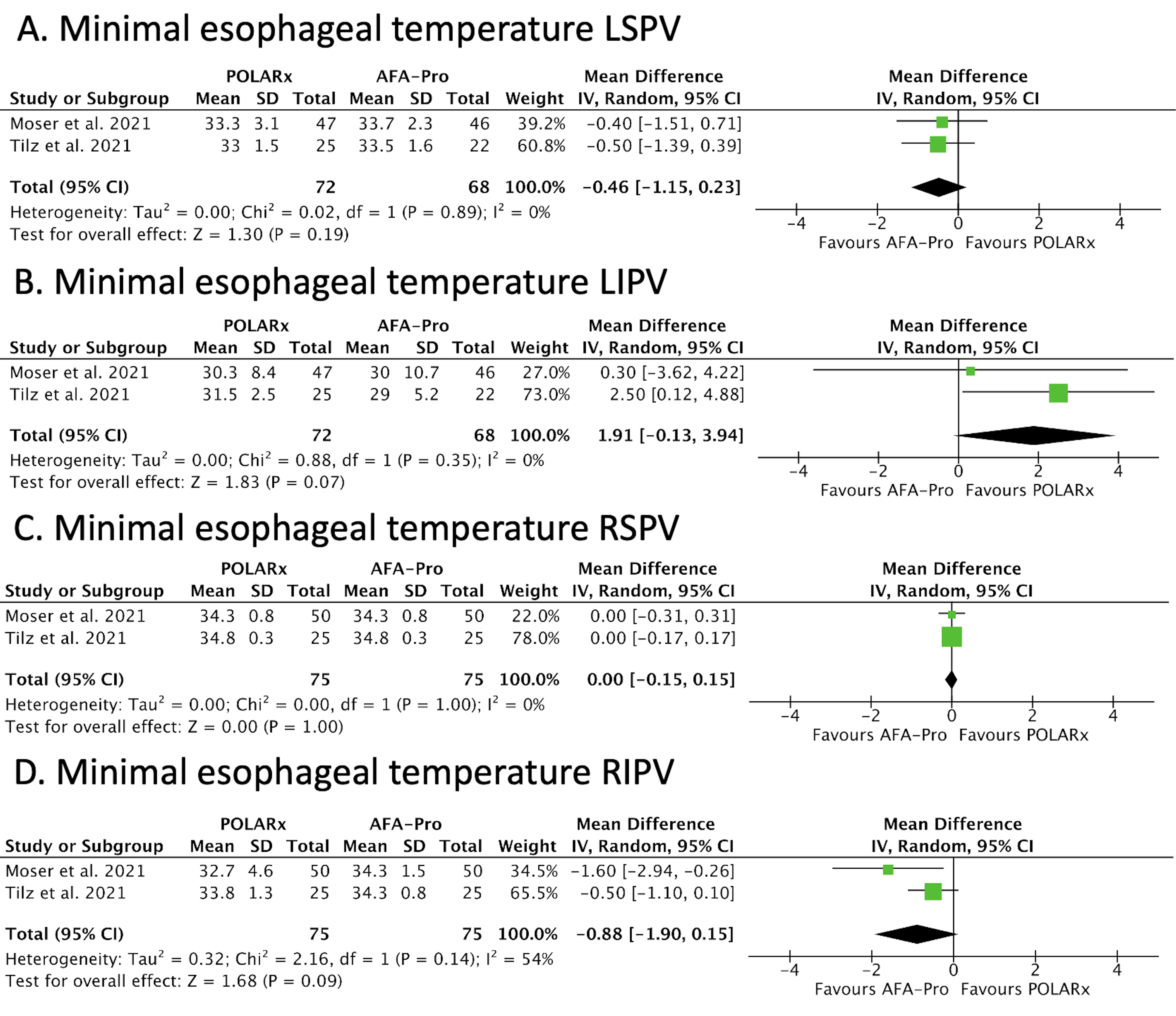


**Supplemental figure** **S5**. Sensitivity analysis. Forest plots of the pooled analysis demonstrating the effect of POLARx versus AFA-Pro. The data are presented as mean, standard deviation and mean difference. The horizontal line is the 95% CI. The diamond shape is the estimate and the confidence interval of the estimate. Abbreviations: LIPV, left inferior pulmonary vein; LSPV, left superior pulmonary vein; RSPV, right inferior pulmonary vein; TTI, time-to-isolation.


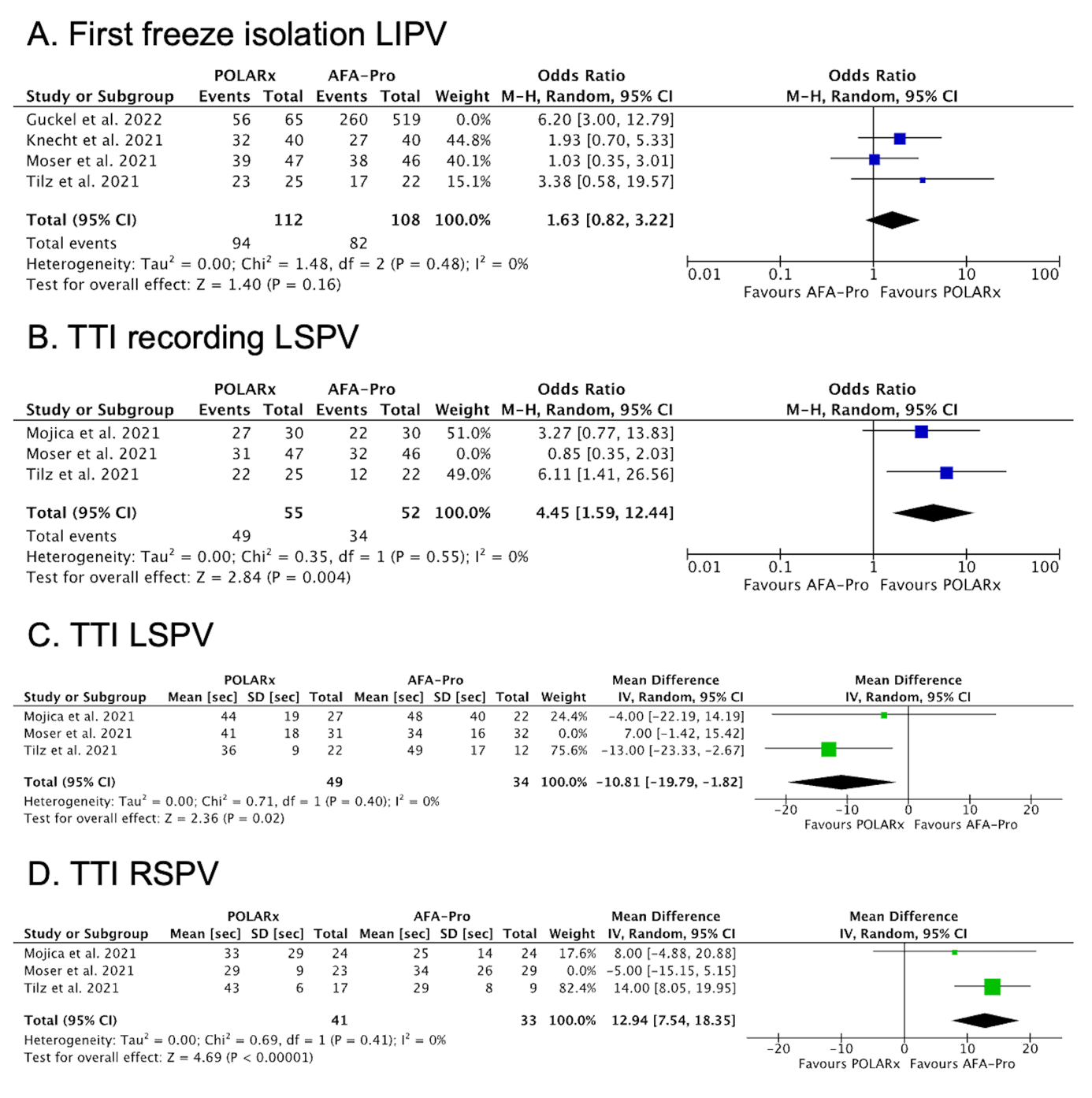


**Supplemental table S1**. Newcastle-Ottawa quality assessment for observational studies

|  | **Selection** | | | | **Comparability** | **Outcome** | | | **Total score** |
| --- | --- | --- | --- | --- | --- | --- | --- | --- | --- |
| **Study** | **Representativeness of the exposed cohort** | **Selection of the non exposed cohort** | **Ascertainment of exposure** | **Outcome not present at start** |  | **Assessment of outcome** | **Adequate follow-up length** | **Adequacy of follow-up** |  |
| Creta et al.^8^ | 🟋 | 🟋 | 🟋 | 🟋 | 🟋 | 🟋 | 🟋 | 🟋 | 8 |
| Guckel et al.^13^ | 🟋 | 🟋 | 🟋 | 🟋 | 🟋 | 🟋 | 🟋 | 🟋 | 8 |
| Knecht et al.^14^ | 🟋 | 🟋 | 🟋 | 🟋 | 🟋 | 🟋 | 🟋 | 🟋 | 8 |
| Kochi et al.^9^ | 🟋 | 🟋 | 🟋 | 🟋 | 🟋 | 🟋 | 🟋 | 🟋 | 8 |
| Mojica et al.^15^ | 🟋 | 🟋 | 🟋 | 🟋 | 🟋🟋 | 🟋 | 🟋 | 🟋 | 9 |
| Moser et al.^16^ | 🟋 | 🟋 | 🟋 | 🟋 | 🟋 | 🟋 | 🟋 | 🟋 | 8 |
| Tilz et al.^10^ | 🟋 | 🟋 | 🟋 | 🟋 | 🟋 | 🟋 | 🟋 | 🟋 | 8 |
| Yap et al.^11^ | 🟋 | 🟋 | 🟋 | 🟋 | 🟋 | 🟋 | 🟋 | 🟋 | 8 |
