# Supplementary material for: Comparison of the acute outcome of two cryoballoon technologies for pulmonary vein isolation: An updated systematic review and meta-analysis
Source: Int J Cardiol Heart Vasc. 2022 Sep 5;42:101115. doi: 10.1016/j.ijcha.2022.101115 (PMC9463571; doi:10.1016/j.ijcha.2022.101115)
Supplement: Appendix B (multimedia component 3, supplementary data 3) [file mmc3.pdf]

Supplemental material

Database searched	via	Years of coverage	Records	Records after duplicates removed
Embase	Embase.com	1971 - Present	986	508
Medline ALL	Ovid	1946 - Present	568	566
Web of Science Core Collection*	Web of Knowledge	1975 - Present	496	64
Cochrane Central Register of Controlled Trials	Wiley	1992 - Present	133	25
Other sources: Google Scholar (maximally 200 top-ranked)			51	36
**Total**			**2234**	**1199**

*Science Citation Index Expanded (1975-present) ; Social Sciences Citation Index (1975-present) ; Arts & Humanities Citation Index (1975-present) ; Conference Proceedings Citation Index- Science (1990-present) ; Conference Proceedings Citation Index- Social Science & Humanities (1990-present) ; Emerging Sources Citation Index (2015-present)

Embase 986

(polarX OR polar-X):ab,ti,kw OR ((('cryoablation'/de) OR (cryoablat* OR cryo-ablat* OR ((cryoballoon* OR cryo-balloon*) NEAR/3 (ablation* OR cathet*)) OR fourth-gen*-cryo* OR 4th-gen*-cryo* OR 4th-CB OR CB4 OR CBG4 OR arctic-front* OR AFA-pro*):ab,ti,kw) AND ('pulmonary vein isolation'/de OR 'pulmonary vein'/de OR (((pulmonar*) NEAR/3 (vein*)) OR PVAI OR PVI):ab,ti,kw) AND [2019-2030]/py) NOT ((animal/exp OR animal*:de OR nonhuman/de) NOT ('human'/exp)) NOT (('juvenile'/exp OR (juvenil* OR adolescen* OR preadolescen* OR youth* OR child* OR schoolchild* OR minors OR teen OR teens OR teenager* OR infan* OR toddler* OR pediatr* OR paediatr* OR puber* OR baby OR babies OR girl* OR boy* OR newborn* OR neonate* OR premature* OR pre-matur* OR kid OR kids OR underag* OR kindergar* OR pubescen* OR prepubesc* OR school* OR preschool* OR highschool* OR suckling OR PICU OR NICU OR PICUs OR NICUs):ab,ti,kw) NOT ('adult'/exp OR (adult* OR elderl* OR man OR men OR woman OR women):ab,ti,kw)) AND [ENGLISH]/lim

Medline 568

(polarX OR polar-X).ab,ti,kf. OR (((Cryosurgery/) OR (cryoablat* OR cryo-ablat* OR ((cryoballoon* OR cryo-balloon*) ADJ3 (ablation* OR cathet*)) OR fourth-gen*-cryo* OR 4th-gen*-cryo* OR 4th-CB OR CB4 OR CBG4 OR arctic-front* OR AFA-pro*).ab,ti,kf.) AND (Pulmonary Veins/ OR (((pulmonar*) ADJ3 (vein*)) OR PVAI OR PVI).ab,ti,kf.) AND 2019:2030.(sa_year).) NOT (exp Animals/ NOT Humans/) NOT ((exp Infant/ OR exp Child/ OR exp Adolescent/ OR (juvenil* OR adolescen* OR preadolescen* OR youth* OR child* OR schoolchild* OR minors OR teen OR teens OR teenager* OR infan* OR toddler* OR pediatr* OR paediatr* OR puber* OR baby OR babies OR girl* OR boy* OR newborn* OR neonate* OR premature* OR pre-matur* OR kid OR kids OR underag* OR kindergar* OR pubescen* OR prepubesc*

OR school* OR preschool* OR highschool* OR suckling OR PICU OR NICU OR PICUs OR NICUs).ab,ti,kf.) NOT (exp Adult/ OR (adult* OR elderl* OR man OR men OR woman OR women).ab,ti,kf.)) AND english.la.

Cochrane 133

(polarX OR polar-X):ab,ti,kw

OR

(with limit set to 2019 onwards)

((cryoablat* OR cryo NEXT ablat* OR ((cryoballoon* OR cryo NEXT balloon*) NEAR/3 (ablation* OR cathet*)) OR fourth NEXT gen* NEXT cryo* OR 4th NEXT gen* NEXT cryo* OR 4th NEXT CB OR CB4 OR CBG4 OR arctic NEXT front* OR AFA NEXT pro*):ab,ti,kw) AND ((((pulmonar*) NEAR/3 (vein*)) OR PVAI OR PVI):ab,ti,kw)

Web of Science 496

TS=((polarX OR polar-X OR (((cryoablat* OR cryo-ablat* OR ((cryoballoon* OR cryo-balloon*) NEAR/2 (ablation* OR cathet*)) OR fourth-gen*-cryo* OR 4th-gen*-cryo* OR 4th-CB OR CB4 OR CBG4 OR arctic-front* OR AFA-pro*)) AND ((((pulmonar*) NEAR/2 (vein*)) OR PVAI OR PVI)))) NOT ((animal* OR rat OR rats OR mouse OR mice OR murine OR dog OR dogs OR canine OR cat OR cats OR feline OR rabbit OR cow OR cows OR bovine OR rodent* OR sheep OR ovine OR pig OR swine OR porcine OR veterinar* OR chick* OR zebrafish* OR baboon* OR nonhuman* OR primate* OR cattle* OR goose OR geese OR duck OR macaque* OR avian* OR bird* OR fish*) NOT (human* OR patient* OR women OR woman OR men OR man)) NOT ((juvenil* OR adolescen* OR preadolescen* OR youth* OR child* OR schoolchild* OR minors OR teen OR teens OR teenager* OR infan* OR toddler* OR pediatr* OR paediatr* OR puber* OR baby OR babies OR girl* OR boy* OR newborn* OR neonate* OR premature* OR pre-matur* OR kid OR kids OR underag* OR kindergar* OR pubescen* OR prepubesc* OR school* OR preschool* OR highschool* OR suckling OR PICU OR NICU OR PICUs OR NICUs) NOT (adult* OR elderl* OR man OR men OR woman OR women))) AND py=(2019-2021) AND LA=(English)

Google Scholar 51

polarX|'polar x' cryoablation|cryoballoon|'pulmonary vein'|'cryo ablation'|'cryo balloon'|'arctic front'|'AFA pro'
